# One-Stage Implant-Retaining Revision for Simultaneous Bilateral Infected Total Knee Arthroplasty: A Report of a Rare Case

**DOI:** 10.1155/2021/8846198

**Published:** 2021-07-13

**Authors:** Nils Wirries, Lars-René Tuecking, Michael Skutek

**Affiliations:** Department of Orthopedic Surgery at Diakovere Annastift, Hannover Medical School, Anna-von-Borries-Str. 1–7, 30625 Hannover, Germany

## Abstract

There is little information on the management of simultaneous infected total knee arthroplasties in the same patient. Although general principles of management for periprosthetic joint infection apply, there might be certain aspects worth to be considered. We present a case of a 78-year-old patient, who was referred in preseptic conditions 10 years following bilateral TKA. The onset of symptoms was less than one week, proposing an acute hematogenous infection. Analysis of joint fluid revealed that both of his TKAs were infected with *Streptococcus sanguinis*. Diagnostic algorithms, surgical principles, and the course of the patients following bilateral revision are being described. The reasons for an implant-retaining procedure with irrigation and debridement including the exchange of the polyethylene liners are being discussed as well as possible principles of management of bilateral periprosthetic joint infections.

## 1. Introduction

The periprosthetic joint infection (PJI) represents one of the most major complications after total knee arthroplasty (TKA) with an incidence of up to 2.0% [1]. Guidelines for management have been proposed and treatment strategies (one-stage vs. two-stage revision) are well established [2, 3]. However, the literature is not very conclusive to follow these general recommendations of infection management in cases of simultaneous PJI. Contemporaneous infections of two major joints are worrisome, especially in an elderly patient population with preseptic conditions and significant comorbidities.

We present a case of an implant retaining bilateral one-stage revision in a 78-year-old patient, who was referred 10 years after the initial TKAs. When admitted, he was in a preseptic condition due to bilateral infected TKA. The management was complicated through comorbidities such as diabetes and congestive heart disease with previous heart surgery.

The patient was informed and agreed for the publication of his case, including all radiographs.

## 2. Case Presentation

The patient received bilateral TKAs (Sigma®, DePuy, Warsaw, Ind.) for end-stage osteoarthritis of both knees, 2009 (left) and 2010 (right). On the left side, he had a previous high tibial osteotomy. In both knees, a posterior stabilized implant was used. The postoperative course was normal, and he returned for scheduled routine follow-up visits (3 months, 1 year, and 5 years). No abnormalities were noted.

10 years after the initial procedures, the patient was referred through the general practitioner to internal medicine for general deterioration of his health status in a clinic with no orthopedic department. He also claimed bilateral knee pain with a sudden onset. Approximately two weeks before, he was diagnosed with bronchitis. The symptoms resolved after a few days without a treatment or further diagnostics. His past medical history was positive for atrial fibrillation, hypertensive heart condition, high blood pressure, hyperuricemia, and mitral and tricuspid regurgitation (2^nd^ degree). His past surgical history included aortocoronary bypass surgery in 2015 and partial thyroidectomy.

At the time of referral to the first non-orthopedic clinic, his general status was reduced, his heart rate was 100 bpm/arrhythmic, and the infection parameters were elevated with a C-reactive protein (CRP) of 20.5 mg/dl (reference value: <0.5 mg/dl) and white blood cells (WBC) of 9.2 tsd/*μ*l (reference value: 3.6–10.5 tsd/*μ*l). Knee effusions were noted bilaterally, and aspiration was performed through the internal medicine service for microbiologic diagnostic. The patient was started empirically on antibiotics with sulbactam and ampicillin (Unacid®) in his preseptic condition. The general condition improved, and he was referred to our orthopedic center after bacteria were proofed in the joint synovial fluid.

At this time, the patient's condition was still generally reduced. The patient was able to walk without supporting devices, but the walking distance was reduced to approximately 500 m under pain in both knees. The clinical examination showed on both sides a gently swollen joint with a right-sided hyperthermia. The range of motion (ROM) was restricted on the right and left sides with 0/0/100° extension/flexion (E/F) under stable conditions. Meanwhile, his CRP has dropped to 6.3 mg/dl. From both initial aspirates, *Streptococcus sanguinis* bacteria were isolated. Under continued empiric antibiotics (Unacid®), repeated aspirates revealed a leucocyte count of 15000/ml with 98% granulocytes in the right knee and a leucocyte count of 14900/ml with 95% granulocytes in the left knee. The fluids remained sterile. The diagnosis of a synchronized, simultaneous infection of both TKAs likely following acute hematogenous cause due to recent bronchitis was established. Prior to surgery, a transesophageal echo showed no valve vegetation. The i.v. antibiotics were changed according to microbiology recommendations to i.v. penicillin and clindamycin.

**Figure 1:**
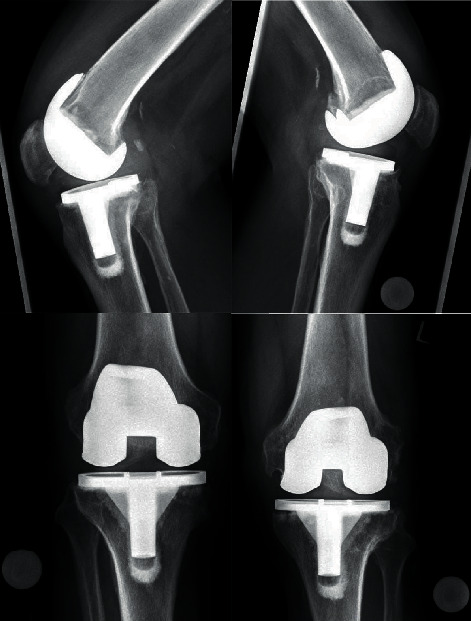
The preoperative diagnostics included radiographs of both knees in 2 planes.

**Figure 2:**
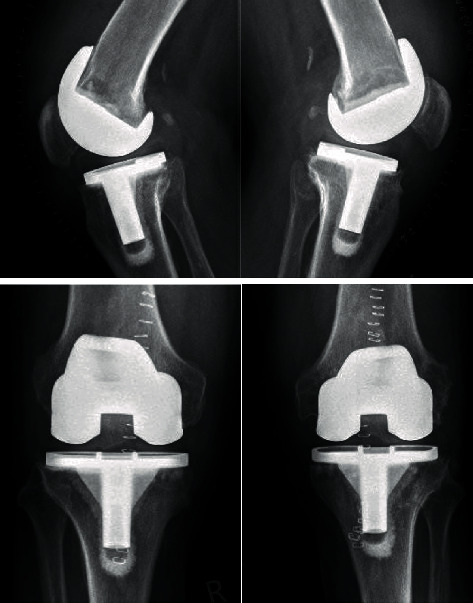
Radiographs in 2 planes directly after the implant-retaining procedure on both sides10 years after TKAs: no hint for loosening and clip suture.

After preoperative diagnostics, the patient was brought in improved conditions to the OR five days after admission (Figure 1). Both knees were revised performing an implant-retaining procedure (Figure 2). The treatment included an irrigation and debridement (I+D) with local application of antiseptics (Serasept®) following radical synovectomy. The polyethylene was changed and a drain was applied. The patient's condition improved directly after surgery, and he was started with full weight bearing and physiotherapy at the first postoperative day. Antibiotics continued with i.v. penicillin and clindamycin for 14 days and were then changed for oral application for additional 3 months. The further course was uneventful with full recovery and no remaining signs of infection in the blood analysis in the follow-up examination 3 and 6 months after surgery so that the decision was made to end the oral antibiotic application to prevent the multimorbid patient from side effects. In detail, the patient showed a ROM of approximately 0/0/120° E/F with stable joint conditions on both sides. The gait ability was increased to 2–3 km with walking devices. In the radiological examination, no hints of loosening or a periprosthetic fracture were found. At the 6-month follow-up examination, both joints had a constant ROM of 0/0/120° under stable conditions. The patient showed an activity level with 6 km cycling each day. However, he described occasional discomfort while going downstairs and hefting. The laboratory findings, especially the CRP (0.36 mg/dl), WBC (6.8 tsd/*μ*l), and interleukin 6 (3.34 pg/ml; reference value: <7.0), were in the normal range. Only the urea was elevated, which the patient clarified by an internal medical doctor. A one-year follow-up was planned, but the patient has not embraced due to the COVID-19 pandemic.

## 3. Discussion

Despite management strategies for PJI after TKA were established, a simultaneous and synchronized infection of bilateral TKA represents a challenge in orthopedic surgery [4]. Due to the fact that literature is not conclusive in these rare cases, the need for further investigations is arising. In the absence of clinical studies, this case report might be an opportunity to discuss this rare occasion.

We presented the case of an implant retaining bilateral one-stage revision in a 78-year-old patient, who suffered from preseptic conditions due to bilateral PJI 10 years after primary TKAs. Although at the time of bronchitis no sputum samples were analyzed, the proofed *Streptococcus sanguinis* represents not a common pathogen for PJI. *Streptococcus sanguinis* is usually found in the oral cavity and causes endocarditis, e.g., after dental treatment [5]. However, a cardiac infection was excluded before revision surgery. Additionally, comorbidities such as diabetes and congestive heart disease represented a further challenge.

Adapted to the staging system for PJI from McPherson et al., the infection represented an acute hematogenous infection (type II) [6]. Systemic factors included diabetes, recent pulmonary bronchitis, age (close to 80 years), active infection (bronchitis), and absolute neutrophil count about 15.000/ml, corresponding type C. Local factors were multiple skin incisions following HTO on the left side, resulting in a PJI type IIC2 in accord with the staging system. The surgical and nonsurgical options were being discussed with the patient. Due to poor results in case of nonsurgical treatment even in cases of PJI with single joint involvement, the surgical procedure was opted [7]. In addition, the patient showed preseptic conditions under relevant cardiopulmonary comorbidities and a disease-related immunodeficiency due to his diabetes. Therefore, we expected a low chance for an improvement of the general state of health with solely i.v. antibiotics. Due to the sudden onset of symptoms comparable to an early PJI, an implant-retaining option was chosen. However, a more aggressive approach with complete implant removal and revision bilateral TKAs in a one- or two-stage setting certainly was discussed. But under consideration of an enlarged narcosis time and prolonged rehabilitation time (especially immobility after implant removal), the risks of cardiovascular complications were expected to be comparatively high. Thus, an implant-retaining procedure was preferred. It is thought to be important presenting this case, in which a comparatively minor surgical procedure resolved the acute problem of the patient, even in a patient with several comorbidities leading further to minor tissue healing capacities. In case of an ongoing infection after an implant-retaining procedure, a two-stage revision would have been performed, if the patient's conditions permitted it. Compared to the successful rate of infection control under antibiotic suppressive therapy (AST) with approximately 67%, a two-stage revision showed a good joint function and an eradication rate of more than 90% [7, 8]. However, if the patient's health status did not allow a surgical debridement with implant removal, the AST would have represented a reasonable alternative.

Another case of a multijoint periprosthetic infection (both knees, right hip) after *Staphylococcus aureus* (MRSA) endocarditis was published by Pina et al. The authors treated the 71-year-old patient with a two-stage implant-removing technique. After resolution, the total hip arthroplasty was replanted first and 6 weeks later both TKAs. At the one-year examination, the patient showed no hints of joint infection under a prophylactic minocycline treatment. The knee function was on both sides marginal restricted with a flexion contracture of approximately 20° [8].

Balkhair et al. presented a 57-year-old female patient with a bilateral PJI 10 years after TKA with Brucella. Due to missing signs of implant loosening, the authors decided to an AST. The PJI resolved, and the patient was free of symptoms or hints of infections three years later at the last time of described follow-up [9].

A synchronized and simultaneous infected bilateral total knee arthroplasty represents a major challenge to the arthroplasty surgeon. Although the general principles of PJI pertain, it seems to be important to be aware that these can also be applied for more severe cases. We classified an acute PJI and believe that our patient benefitted from the decision to an implant-retaining revision, especially regarding his comorbidities. Up to now, no complications or further surgeries were required. In summary, implant-retraining revision might be an alternative treatment option in a selected patient clientele, like the one just presented.

## Data Availability

The datasets generated during and/or analyzed during the current study are not publicly available due to data privacy, but are available from the corresponding author on reasonable request.
